# Differentiated Effects of Secondary Metabolites from *Solanaceae* and *Brassicaceae* Plant Families on the Heartbeat of *Tenebrio molitor* Pupae

**DOI:** 10.3390/toxins11050287

**Published:** 2019-05-22

**Authors:** Paweł Marciniak, Angelika Kolińska, Marta Spochacz, Szymon Chowański, Zbigniew Adamski, Laura Scrano, Patrizia Falabella, Sabino A. Bufo, Grzegorz Rosiński

**Affiliations:** 1 Department of Animal Physiology and Development, Institute of Experimental Biology, Faculty of Biology, Adam Mickiewicz University in Poznań, 61-614 Poznań, Poland; kolinska.angelika@gmail.com (A.K.); marta.spochacz@amu.edu.pl (M.S.); szyymon@amu.edu.pl (S.C.); ed@amu.edu.pl (Z.A.); rosin@amu.edu.pl (G.R.); 2 Electron and Confocal Microscope Laboratory, Faculty of Biology, Adam Mickiewicz University in Poznań, 61-614 Poznań, Poland; 3 Department of European and Mediterranean Cultures, University of Basilicata, 75100 Matera, Italy; laura.scrano@unibas.it; 4 Department of Sciences, University of Basilicata, 85100 Potenza, Italy; patrizia.falabella@unibas.it (P.F.); sabino.bufo@unibas.it (S.A.B.); 5 Department of Geography, Environmental Management & Energy Studies, University of Johannesburg, Auckland Park Kingsway Campus, Johannesburg 2092, South Africa

**Keywords:** plant secondary metabolites, glycoalkaloids, insect heart, beetles, insect, *Tenebrio molitor*

## Abstract

The usage of insects as model organisms is becoming more and more common in toxicological, pharmacological, genetic and biomedical research. Insects, such as fruit flies (*Drosophila melanogaster*), locusts (*Locusta migratoria*), stick insects (*Baculum extradentatum*) or beetles (*Tenebrio molitor*) are used to assess the effect of different active compounds, as well as to analyse the background and course of certain diseases, including heart disorders. The goal of this study was to assess the influence of secondary metabolites extracted from *Solanaceae* and *Brassicaceae* plants: Potato (*Solanum tuberosum*), tomato (*Solanum lycopersicum*), black nightshade (*Solanum nigrum*) and horseradish (*Armoracia rusticana*), on *T. molitor* beetle heart contractility in comparison with pure alkaloids. During the in vivo bioassays, the plants glycoalkaloid extracts and pure substances were injected at the concentration 10^−5^ M into *T. molitor* pupa and evoked changes in heart activity. Pure glycoalkaloids caused mainly positive chronotropic effects, dependant on heart activity phase during a 24-h period of recording. Moreover, the substances affected the duration of the heart activity phases. Similarly, to the pure glycoalkaloids, the tested extracts also mainly accelerated the heart rhythm, however *S. tuberosum* and *S. lycopersicum* extracts slightly decreased the heart contractions frequency in the last 6 h of the recording. Cardioacceleratory activity of only *S. lycopersicum* extract was higher than single alkaloids whereas *S. tubersoum* and *S. nigrum* extracts were less active when compared to pure alkaloids. The most cardioactive substance was chaconine which strongly stimulated heart action during the whole recording after injection. *A. rusticana* extract which is composed mainly of glucosinolates did not significantly affect the heart contractions. Obtained results showed that glycoalkaloids were much more active than glucosinolates. However, the extracts depending on the plant species might be more or less active than pure substances.

## 1. Introduction

Two groups of assimilates stand out in the processes of biosynthesis occurring in plants: basic substances and products of secondary metabolism [1]. Primary metabolites (carbohydrates, proteins, and fats) are found in all plants where they perform basic physiological functions. Secondary substances occur only in specific systematic groups, that means they do not perform the functions necessary for life. However, they often have the character of physiologically active compounds, that makes them applicable in different fields, for instance in medicine or agriculture.

A broad spectrum of physiological activity is demonstrated by alkaloids [2]. They have been proved to exhibit antioxidant, anti-inflammatory, anti-aggregation, hypocholesteric, immunostimulant or anticancer properties. Many of them belong to the group of compounds modifying or normalizing the activity of the heart. The mode of action is often connected with the ability to control calcium channels, similarly to synthetic substances/drugs [3].

Alkaloids are produced by different plants species such as *Papaveraceae*, *Fabaceae*, *Ranunculaceae* or *Solanaceae* plant families or lower plants such as forks or creaks [4]. They are usually basic nitrogen-containing organic compounds, mainly synthesized from amino acids. Substrates for about five thousand of this type of compound are three protein amino acids: phenylalanine, lysine and tryptophan.

**Figure 1:**
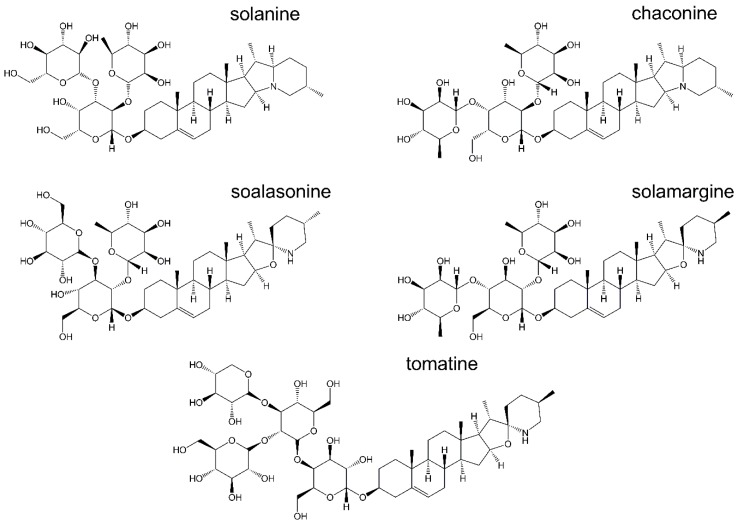
Structures of major potato, black nightshade and tomato glycoalkaloids.

Nowadays, emerging interest is put on *Solanaceae* alkaloids. They are mainly steroidal glycoalkaloids (GAs)-glycosidic derivatives of nitrogen-containing steroids that are produced in more than 350 plant species [5]. The major representatives of this glycoalkaloid family are α-solanine and α-chaconine in potato plants (*Solanum tuberosum* L.), solasonine and solamargine in black nightshade (*Solanum nigrum* L.) or α-tomatine and dehydrotomatine in tomato plants (*Solanum lycopersicum* L.) [6,7] (Figure 1). They have been shown to possess a wide spectrum of biological activity at the molecular, cellular and organismal levels [6,8].

Insects are suitable models for the study of various active compounds, including plant derived substances. They have a short life cycle and are easy to grow. There are also reports suggesting possible similarities in the mechanisms of action of various active substances in relation to vertebrate and invertebrate animals [9]. Increasingly, they are used in various toxicological or pharmacological researches [9,10].

Previous investigation has shown that *Solanaceae* GAs or *Solanaceae* plant extracts are able to influence the heart activity of *Zophobas atratus* Fab. beetle [11]. Chosen GAs irreversibly stopped *Z. atratus* hearts in in vitro conditions but surprisingly stimulated the activity in in vivo bioassays, alternating the duration of phases in the heart cycle. Thus, further experiments were needed to explain whether GAs are cardiotoxic or just cardioregulatory. Using a different model beetle species *Tenebrio molitor*, here, it is presented further evidence that *Solanaceae* GAs differentially affect the insect heart activity. Furthermore, a different group of plant secondary metabolites have been tested—glucosinolates (GLSs) present in *Armoria rusticana* extract [7], in order to check whether they possess cardioregulatory activity.

## 2. Results

### 2.1. Heart Rhythm of the Tenebrio molitor Pupae

**Figure 2:**
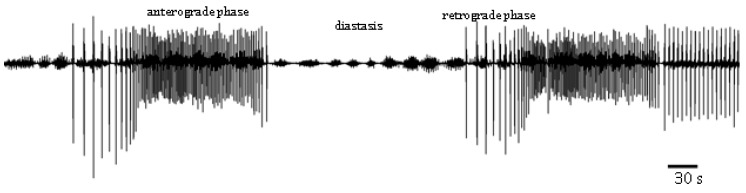
Myocardiograms of the *Tenebrio molitor* pupal heartbeat registered under control conditions.

**Figure 3:**
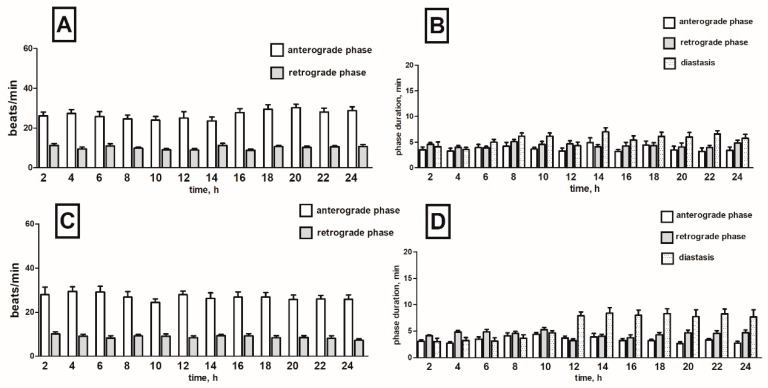
Changes in the heart rate (**A**,**C**) and in the duration of anterograde, retrograde phases and diastase in the cardiac cycle (**B**,**D**) of *T. molitor* pupae in 2-h intervals, registered without injection (**A**,**B**) and after injection of physiological saline (**C**,**D**). Statistically significant differences (*p* ≤ 0.05, Mann-Whitney test) when compared to control are indicated with an asterisk, *n* ≥ 10.

Cardiac contractile activity of *T. molitor* intact pupae was examined by a non-invasive optoelectronic technique. In order to detect circadian changes in the heart rhythm, 24-h recordings were conducted. The recordings indicated that the heart rhythm of one-day-old pupae is complex. A constant pattern in the work of this organ was manifested by regular alternations of: (1) forward orientated, fast—anterograde phase, (2) backward orientated, slow—retrograde phase, and (3) no contraction phase—diastasis. These phases, apart from the direction of propagation of peristalsis contractions wave, differed also in the frequency of contractions of myocardium and their duration (Figure 2). Average heart contraction frequency of intact *T. molitor* pupae was 26/min in anterograde phase and 10/min in retrograde phase (Figure 3A). The average duration of each phase was 3 min 40 s for anterograde phase, 4 min 20 s for retrograde phase and 5 min 30 s for the diastasis (Figure 3B). The length of the phases depends on the time of recording (day or night). The injection of physiological saline caused minor effects in the heartbeat (Figure 3C) and had no effects in the duration of the phases (Figure 3D). These effects were not statistically significant and thus were not taken under consideration during the experiments with tested substances.

### 2.2. Effects of Potato Glycoalkaloids on the Heart Rhythm of T. molitor Pupae

#### 2.2.1. Pure Alkaloids

##### α-Solanine

**Figure 4:**
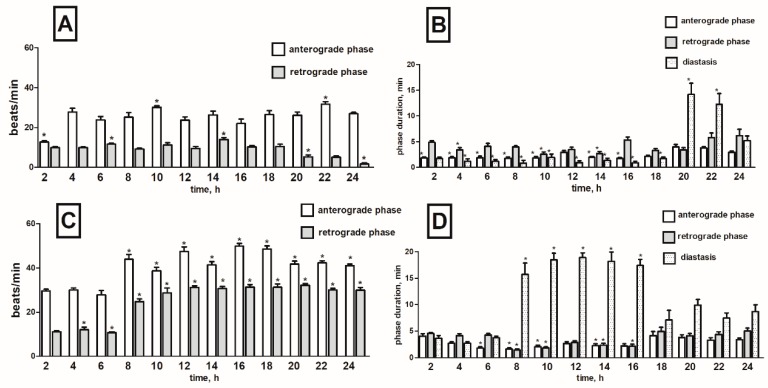
Changes in the heart rate (**A**,**C**) and in the duration of anterograde, retrograde phases and diastase in the cardiac cycle (**B**,**D**) of *T. molitor* pupae in 2-h intervals, registered after injection of α-solanine (1 × 10^−5^ M) (**A**,**B**) and α-chaconine (1 × 10^−5^ M) (**C**,**D**). Statistically significant differences (*p* ≤ 0.05, Mann-Whitney test) when compared to control are indicated with an asterisk, *n* ≥ 10.

Injection of α-solanine caused ambiguous effects which strongly depended on time after application. In the first two hours, we noticed strong cardioinhibitory effects in the anterograde phase with the frequency of heart contraction decreasing by 47% when compared to the control (physiological saline injections). The effect was evident in just two first hours after injection. The negative chronotropic effect in this phase was also recorded in the 6th and 12th hour of the recording (decreasing by 20 and 16%) but the results were not statistically significant (Figure 4A). Otherwise, in the 10th and 22nd hour of recording, the frequency of the heart contractions in anterograde phase significantly increased (both by 22%) (Table S1). During the first part of recording (4th–18th h), an increase in the heart rate of 18% on average was observed in retrograde phase. A clear chronotropic-positive effect occurred in 6th and 14th hour of registration (up by 38 and 49% respectively) (Table S1). Nevertheless, in the final hours of recording (20th–24th h), cardioinhibitory effects of α-solanine were observed. The frequency of contraction 20, 22 and 24 h after injection was reduced by 38, 38 and 77%, respectively when compared to the control (Figure 4A).

Injection of α-solanine altered duration of phases of pupal heart rhythm. Shortening of fast anterograde phase by 1 min and 39 s on average was observed between the 2nd and 16th hour, later the effect silenced (Figure 4B). Changes in retrograde phase varied depending on time after compound application. Moreover, α-solanine caused reduction of diastase duration between the 2nd and 16th hour of the recording period by an average of 4 min (Table S2). In the next hours, the effect weakened and finally in the 20th and 22nd hour of registration, strong elongation of the diastase with an average of 4 min 40 s was observed (Figure 4B).

##### α-Chaconine

Injection of α-chaconine induced an increase in the contraction frequency of 66% on average, in the anterograde phase, and 200% in the retrograde phase during the entire registration period (Table S1). The highest-positive chronotropic effect in fast phase was observed at 16 and 18 h after compound injection (Figure 4C). In this period, the frequency of contractions increased by 86%. In slow phase, increased contraction frequency was noticed from the 4th to the 24th hour of registration, and it was 210% higher on average than in the control (Figure 4C). Application of α-chaconine shortened the anterograde phase between the 6th and 16th hour during the recording by 1 min and 32 s on average. Retrograde phase was reduced by an average of 1 min and 46 s between the 8th to 16th hour after injection (Table S2). The opposite effect was observed during the diastase. α-Chaconine injections caused a significant prolongation of this phase between the 8th and 16th hour of recording, by 10 min and 50 s on average (Figure 4D).

#### 2.2.2. *S. tuberosum* Extract

**Figure 5:**
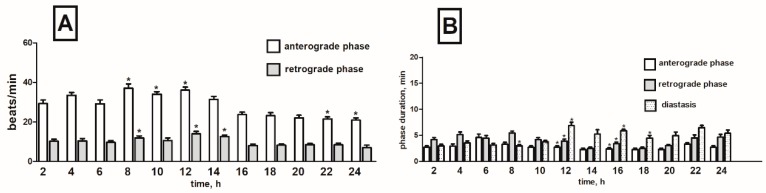
Changes in the heart rate (**A**) and in the duration of anterograde, retrograde phases and diastase in the cardiac cycle (**B**) of *T. molitor* pupae in 2-h intervals, registered after injection of potato extract (1 × 10^−5^ M). Statistically significant differences (*p* ≤ 0.05, Mann-Whitney test) when compared to control are indicated with an asterisk, *n* ≥ 10.

In order to ascertain whether potato (*S. tuberosum)* leaf extract containing known ratio of α-chaconine/α-solanine and some other minor GAs caused similar cardiotropic effects as pure substances, the pupae were injected with the potato extract prepared as described above. Application of potato extract caused, in the initial hours of registration (2nd–14th h), a weak increase in frequency of heart contractions in both phases by an average of 21% in the anterograde phase and 23% in retrograde phase. In the later hours of the registration (16th–24th h) frequency decreased by 9% in the anterograde and 3% in retrograde phases (Table S1). In the fast phase the highest positive chronotropic effect was recorded at the 10th hour after injection (by 40% compared to the control). In the slow phase the strongest increase occurred at the 12th hour of registration (by 67% compared to control) (Figure 5A). The injection of the potato extract slightly shortened the duration of the anterograde phase between 8th and 20th hours of the registration (by 44 s on average). In the retrograde phase differentiated changes were noticed depending on the examined interval in the circadian cycle (Table S2). The duration of diastase between the 8th and 24th hour was reduced by an average of 1 min and 52 s (Figure 5B).

### 2.3. Effects of Black Nightshade Glycoalkaloids on the Heart Rhythm of T. molitor Pupae

#### 2.3.1. Pure Alkaloids

##### Solamargine

**Figure 6:**
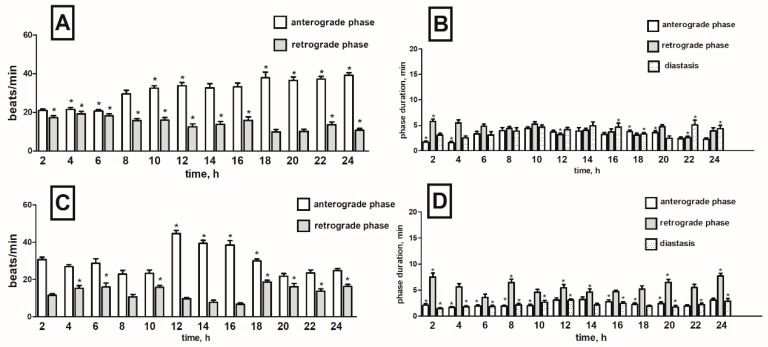
Changes in the heart rate (**A**,**C**) and in the duration of anterograde, retrograde phases and diastase in the cardiac cycle (**B**,**D**) of *T. molitor* pupae in 2-h intervals, registered after injection of solamargine (1 × 10^−5^ M) (**A**,**B**) and solasonine (1 × 10^−5^ M) (**C**,**D**). Statistically significant differences (*p* ≤ 0.05, Mann-Whitney test) when compared to control are indicated with an asterisk, *n* ≥ 10.

Two major *S. nigrum* glycoalkaloids are solamargine and solasonine. Solamargine injection resulted in a decrease of the contractions frequency in the anterograde phase of initial part of the recording time (2nd–6th h) by 30% on average (Table S1). In the following hours (8th–24th h), an increase in contractile activity in this phase by 84% on average occurred. In the slow phase, contractions frequency increased by 56% on average throughout the whole period of registration (Figure 6A).

Injection of solamargine evoked slight shortening of the duration of the fast phase in the first 4 h of registration by 1 min and 4 s on average. Following hours of anterograde phase duration did not differ significantly from the control values. In addition, under the influence of solamargine, prolongation of the retrograde phase in the 2nd hour of recording by 1 min and 30 s was observed. In the next hours, the effect weakened and finally shortening in the 18th and 22nd hour by 1 min 10 s and 1 min and 50 s, respectively, was observed (Table S2). The length of retrograde phase in other intervals did not differ significantly from the control values. The observed diastasis duration shortened on average by 2 min 15 s throughout the whole circadian cycle (Figure 6B). 

##### Solasonine

After solasonine application, an increase in the frequency of contractions in the fast phase between the 12th and 18th hour of registration by 43% on average, in relation to the control, was observed (Table S1). In the retrograde phase, contractile activity increased by 43% on average, throughout the period of registration. The highest positive chronotropic effect was recorded in the 18th and 24th hour when the frequency of contractions was two times higher than in the control (Figure 6C). 

In the pupae treated with solasonine, we noticed a shortened duration of the anterograde phase by 1 min 20 s on average throughout the whole registration period. In the retrograde phase, varied changes in its duration were observed that depended on the analysed interval of the circadian cycle. Significant changes were noted during the phase of diastase (Table S2). Solasonine caused the shortening of this phase by 3 min 55 s on average throughout the registration period (Figure 6D).

#### 2.3.2. *S. nigrum* Extract

**Figure 7:**
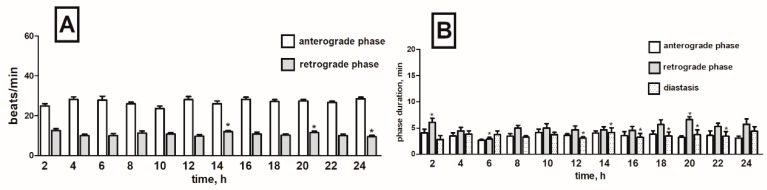
**Changes** in the heart rate (**A**) and in the duration of anterograde, retrograde phases and diastase in the cardiac cycle (**B**) of *T. molitor* pupae in 2-h intervals, registered after injection of *S. nigrum* extract (1 × 10^−5^ M). Statistically significant differences (*p* ≤ 0.05, Mann-Whitney test) when compared to control are indicated with an asterisk, *n* ≥ 10.

Changes in the cardiac anterograde phase after injection of black nightshade (*S. nigrum*) extract were minor and showed different changes depending on the considered time interval in the circadian cycle (Table S1). During the retrograde phase, an increase in the frequency of contractions by 19% on average throughout the whole period of registration was observed (Figure 7A). 

Similarly, injection of black nightshade extract did not cause significant changes in the duration of the fast phase when compared to control values in each recording interval. During the slow phase, a clear reduction occurred in the duration of this phase at the 6th hour by 2 min 2 s and elongation at 2nd and 20th hour by 1 min 50 s and 1 min 43 s, respectively. At other intervals, the length of the retrograde phases was comparable to control values. The extract of black nightshade significantly influenced the duration of diastase but the observed effects were delayed and appeared between the 12th and 24th hour of recording (Table S2). In that period, the resting phase was shortened by an average of 4 min and 10 s (Figure 7B).

### 2.4. Effects of Tomato Glycoalkaloids on the Heart Rhythm of T. molitor Pupae

#### 2.4.1. Synthetic α-Tomatine

**Figure 8:**
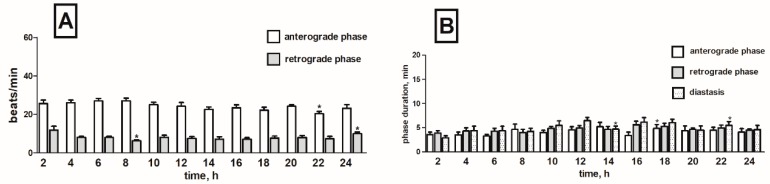
Changes in the heart rate (**A**) and in the duration of anterograde, retrograde phases and diastase in the cardiac cycle (**B**) of *T. molitor* pupae in 2-h intervals, registered after injection of α-tomatine (1 × 10^−5^ M). Statistically significant differences (*p* ≤ 0.05, Mann-Whitney test) when compared to control are indicated with an asterisk, *n* ≥ 10.

The major glycoalkaloid in *S. lycopesicum* is *α*-tomatine; thus, it was tested in order to evaluate the effect of this substance to the heart rhythm of the pupae. Injection of α-tomatine caused a small decrease in the contractile activity of the heart in the anterograde phase with an average of 10% over the entire registration period and a decrease in the frequency of contractions of this organ in the retrograde phase between the 4th and 22nd hour with an average of 13% (Figure 8A). 

α-Tomatine prolonged the duration of the anterograde phase in the second half of the circadian cycle (between 12th and 24th hour) by 1 min on average for each interval compared to the control. The duration of retrograde phases throughout the registration period varied from one period to the next (Table S2). The duration of diastasis under the influence of α-tomatine, similarly to anterograde phase, was prolonged in the second half of the recording period (between 12th and 24th hour) by 2 min 33 s on average (Figure 8B).

#### 2.4.2. *S. lycopersicum* Extract

**Figure 9:**
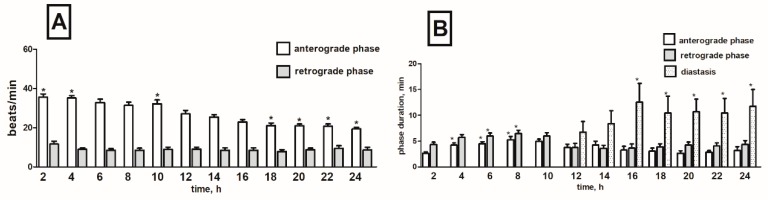
Changes in the heart rate (**A**) and in the duration of anterograde, retrograde phases and diastase in the cardiac cycle (**B**) of *T. molitor* pupae in 2-h intervals, registered after injection of *S. locypersicum* extract (1 × 10^−5^ M). Statistically significant differences (*p* ≤ 0.05, Mann-Whitney test) when compared to control are indicated with an asterisk, *n* ≥ 10.

After the injection of tomato (*S. lycopersicum*) leaf extract, an increase in the frequency of contractions in the anterograde phase between the 2nd and 10th hour of recording, by 26% on average, was observed. In subsequent periods (12th–24th h), the contractile activity of the heart decreased by 15% on average in comparison to the control (Table S1). The response of the heart to the injection of the tomato extract in the retrograde phase was slight and showed varied changes depending on the time interval in the circadian cycle of recording (Figure 9A). 

The extract obtained from tomato extended the duration of the anterograde phase in time between the 4th and 8th hour of the registration period by 1 min and 10 s on average. In addition, the duration of the retrograde phase increased by 1 min and 22 s average in the period between 6th and 8th hour of registration and shortened by 20 s on average in the second half of registration (between 14th and 24th hour). The duration of diastasis in the initial hours of the registration (2nd–10th h) decreased by an average of 2 min and 48 s and was not present (Table S2), while at later intervals (16th–24th h) increased by an average of 3 min and 4 s (Figure 9B).

### 2.5. Effects of Horseradish Glucosinolates on the Heart Rhythm of T. molitor Pupae

**Figure 10:**
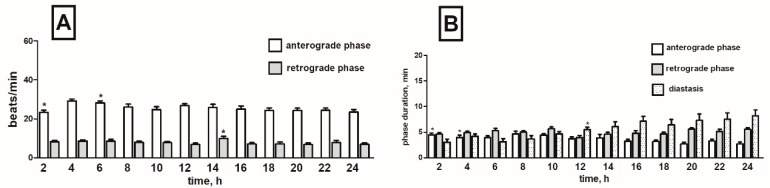
Changes in the heart rate of *T. molitor* pupae in 2-h intervals registered after injection of *A. rusticana* extract in concentration 1 × 10^−5^ M (**A**) together with changes in the duration of anterograde, retrograde phases and diastasis in the cardiac cycle of these pupae after injection of this extract (1 × 10^−5^ M) (**B**). Statistically significant differences (*p* ≤ 0.05, Mann-Whitney test) when compared to control are indicated with an asterisk, *n* ≥ 10.

When the pupae were treated with the horseradish extract, a slight decrease in the frequency of contractions over the entire registration period by 5% on average in the anterograde phase and by 10% in the retrograde phase was noticed (Figure 10A). 

Horseradish extract did not significantly affect the duration of individual phases in the heart cycle. Slightly extended duration time of the anterograde phase in the range from the 2nd to 4th hour of registration was observed (Table S2). Duration of individual retrograde phases was comparable to the corresponding values in the control. During the second half of the recording period (between the 12th and 22nd hour), the diastase was shortened by 1 min on average in each interval (Figure 10B).

## 3. Discussion

Insects are increasingly used as model organisms in toxicological, pharmacological, genetic or biomedical research. In addition to economic aspects, such as low farming costs and a short development cycles, the arguments for using insects in experiments are the similarities which they exhibit in relation to mammals [9,12]. Functional analogies at the molecular and cellular level between the myocardium of the insect and the mammalian heart were shown in the research on the fruit fly *D. melanogaster*, among others, by Bier and Bodmer [13] and Ocorr et al. [14]. Nowadays, other insects, such as locusts (*L. migratoria*), stick insects (*B. extradentatum*), moths (*B. mori)* or beetles (*T. molitor*) are used more and more often to evaluate the action of various bioactive substances, as well as to analyse the basis and the course of some diseases [15,16,17]. 

The research conducted so far has shown that the effects of different alkaloids on animal circulatory systems manifest, among others, through a significant increase or decrease of blood pressure, bradycardia, reduced respiratory activity, and haemolysis. It is believed that these changes are the result of inhibition of acetylcholinesterase activity, increase of cell membrane permeability, as well as disruption of steroid metabolism [18]. However, no detailed data is available on the activity of *Solanaceae* GAs on heart activity. To the best of our knowledge, there are only two published reports that study cardiotropic properties of GAs on vertebrates. Bergers and Alink [19] examined effects of *α*-solanine and *α*-tomatine in cultured beating rat heart cells and observed ceased beating within a few minutes after the application of *α*-solanine (80 μg/mL) or *α*-tomatine (20 μg/mL). What is interesting, is that at lower concentrations, both compounds increased the contraction frequency of the heart cells. That indicates the high dependence of observed effects on the dose. Different GAs have also been tested for cardiotropic activities on the isolated frog heart. This study showed cardio-stimulatory properties of these compounds which are directly related to the kind of sugars contained in the GAs [20]. Introduction of a new in vivo insect model (pupae of beetles) gave new data on the potent cardiotropic activity of different GAs and GLSs. It also proved, that myocardium of insects can be successfully used to evaluate the cardioactive properties of various active substances, including plant derived molecules, in relatively fast, cheap and ethically more acceptable tests. 

Previous research showed that potato GAs applied as extract caused a decrease in the heart contraction in *Z. atratus* beetles in in vitro conditions while two other tested species (*T. molitor* and *Leptinotarsa decemlineata*) were resistant to these extracts [21]. Further detailed studies showed that potato and tomato extracts applied on *Z. atratus* semi-isolated heart caused irreversible cardiac arrests, while an extract from black nightshade caused fast but reversible arrests [11]. *S. nigrum* extract showed also reversed inhibition of heart activity when applied on the *T. molitor* adult heart in vitro. The reversible few-second heart arrest was observed after the application of extract at a 1% concentration [22]. In *Z. atratus*, pure commercial GAs (*α*-tomatine, α-chaconine, α-solanine, solamargine, and solasonine) caused similar but less evident effects compared with extracts, whereas in *T. molitor* no significant alterations after in vitro application of pure solasonine and solamargine were observed [22]. These results supported the hypothesis that the bioactivity of tested compounds depended on their structure and suggested the existence of synergistic interactions with the extracts [11]. Interestingly, injection of tomato and potato extracts in 1-day-old pupae (in vivo studies) of *Z. atratus,* produced completely opposite effects, inducing reversible positive chronotropic effects and decreased the duration of both phases (anterograde and retrograde) of heart contractile activity [11]. Different results of in vivo and in vitro studies suggest indirect effects of GAs, through alterations of insect physiology that are not known yet. These results are partially in tune with results obtained in the present study. Most tested extracts and single substances exerted a cardiostimulatory effects (chronotropic positive effects). Only *S. nigrum* extract and *A. rusticana* extract showed no-significant effects on the heart cycle of *T. molitor* pupae. This proves that cardiostimulatory effects of GAs are not species-specific in vivo. This was also confirmed when single synthetic substances were tested. Among six tested GAs only tomatine did not produce some effects. However, on the contrary to the results obtained on *Z. atratus,* the effects produced by *S.tuberosum*, *S. nigrum*, and *S. lycopresicum* extracts were similar or even smaller than that of single substances. This indicates that in in vivo studies synergistic interactions of major molecules are not so important for the specific effect. The reason for this is not known so far and needs further explanation. Perhaps, the detoxifying mechanisms of the organism may be activated more by the presence of a variety of minor toxic agents in extracts or they reveal an antagonistic effect on the activity to the heart. Next, some other, minor substances present in the extract, may additionally affect the effect of major substances. 

All tested substances and extracts showed no-cardiotoxic activity. The most cardioactive turned out to be chaconine. The chronotropic positive effect caused by this molecule was the strongest however, it also strongly affected heart cycle phases, increasing the duration of diastasis. These results are different than those obtained on the frog heart [20]. *α*-Tomatine showed the most potent cardiostimulatory activity on the frog heart in comparison to *α*-chaconine and *α*-solanine. Thus, GAs may reveal species-specific effects. However, our results support the hypothesis that the cardioactivity of *Solanaceae* GAs depends on the nature of the aglycone and the number of sugar moieties rather than on the kinds of sugars or their stereochemical configuration in the molecule [20]. 

Lack of cardiotoxicity of tested substances is a quite promising and interesting result and gives new possible direction to use GAs as cardioregulatory factors supporting for example therapies with synthetic molecules. This is also quite surprising because research performed so far showed cytotoxic properties of *Solanaceae* GAs on the isolated heart cells [19]. Possibly, in vitro models give different results compared with in vivo bioassays due to the direct interaction between the tested substances and the cells. Also, research performed on different insect species showed that different GAs or GAs-containing plant extracts alter the health of individuals. For example, *S. nigrum* alkaloids and *A. rusticana* GLSs affected the development and caused imago malformations of model species *D. melanogaster* [23], whereas tomato and potato GAs and their extracts interrupted the functioning of the fat body cells of the moth *Spodoptera exigua* [24] and enzymes’ activity in *Galleria mellonella* moths [25]. Thus far, glycoalkaloids have been considered as potent bioinsecticides and cytotoxic agents rather than therapeutic molecules. On the other hand, *Solanaceae* GAs have been shown to have anticancer activity [26,27]. However, research obtained in this study showed new pharmacological possibilities of the tested plant-derived substances in the modulation of heart muscle activity. The results also indicate that the method of application may play a crucial role in the mode of action and switching from therapeutic to toxic activity. Therefore, further, more detailed analyses of the biological activity of plant-derived substances are highly likely to bring new, valuable data and suggest their future applications. This conclusion is strongly supported by the increasing trend of scientific interest in the activity of GAs and GLs, observed worldwide [28,29].

## 4. Materials and Methods

### 4.1. Insects

*T. molitor* L. (Coleoptera: Tenebrionidae) 1-day-old pupae were obtained from a colony maintained at the Department of Animal Physiology and Development, Adam Mickiewicz University, Poznań, Poland, according to the procedure described previously [30].

### 4.2. Chemical Standards

Pure solamargine (97.5%) and solasonine (98.3%) were purchased from Glycomix (Compton, UK), pure *α*-chaconine (≥95%) and *α*-solanine (≥95%) were obtained from Lab Service Analytica (Bologna, Italy), while hydrate *α*-tomatine was supplied by Sigma–Aldrich (Taufkirchen, Germany). Commercial *α*-tomatine contained dehydrotomatine as an impurity (*α*-tomatine:dehydrotomatine 10:1 *w*/*w*). Standards were dissolved in physiological saline for beetles (274 mM/L of NaCl, 19 mmol/L of KCl, 9 mmol/L of CaCl_2_) to obtain 10^−3^ M concentration. Samples were stored at −20 °C. Immediately before use, the dilutions of tested compounds were prepared to the desired concentration.

### 4.3. Plant Material and Extracts Preparation

The extracts used for bioassays were prepared from plants grown in Bari, southern Italy, according to the previously described procedure [31]. Potato *S. tuberosum* L. and tomato *S. lycopersicum* Mill. leaves were collected before the fruiting. Green unripe fruits were harvested from the black nightshade. Collected plant material was immediately frozen to suppress the ripening process. Before the extraction the leaves and fruits were stored at −20 °C. The extraction of alkaloids was performed with 1% aqueous acetic acid solution. Each 1.5 g of sample of the material was placed in 20 mL of extraction solution. In order to enhance the contact of plant tissues with the solution, the suspension was stirred for about 2 h, and then centrifuged at 6000 rpm for about 30 min. After extraction the supernatant was lyophilized. *A. rusticana* extract was prepared according to previously described protocol [32]. Lyophilized samples were dissolved in physiological saline for beetles containing 0.1% of acetic acid to obtain 1 × 10^−3^ M concentration and stored at −20 °C before using. Immediately before use, the dilutions of test compounds were prepared to the desired concentration (10^−5^ M). Stock solutions of extracts were obtained at a concentration of 1 × 10^−3^ M for their main component metabolite (*α*-chaconine, *α*-tomatine, and solamargine, respectively). The voucher specimens of all plants were deposited at Herbarium Lucanum (HLUC, Potenza, Italy) with the ID Codes: 5809 *S. tuberosum*, 4433 *S. lycopersicum*, 2320 *S. nigrum* and 9197 *A. rusticana*.

### 4.4. In Vivo Heart Bioassay

The experiments were performed according to the optoelectronic technique performed previously by Slama and Rosiński [15] and Chowański and Rosiński [33]. In brief, it uses alternating visible red light (1–5 kHz) emitted from common light emitting diodes. The light beam is carried from the diode to the dorsal pericardial region of the pupa through a thin outgoing optic fibre. An associated incoming optic fibre collects the reflected pulse light (modulated mainly by movements of the heart and slightly also by other organs) and delivers it to a phototransistor at the opposite end of the fibre. Rarely, during the registration, very high amplitude of recorded contractions was observed, which corresponded to the movement of other organs or the whole body—respiratory movements. These movements were not assessed. The injection of the pupae was performed using a Hamilton syringe, puncturing the cuticle between the 2nd and 3rd segments of the abdomen, on the dorsal side. The needle was inserted towards the head, to a depth of about 3 mm tangentially to the surface of the body. The tested solutions were applied in a volume of 2 μL. For the injection, alkaloids with a concentration of 1 × 10^−5^ M were used, which corresponds with the final concentration in haemolymph of 10^−6^ M. The needle of the syringe was left for a few seconds in the body of the insect, which accelerated the clotting of the hemolymph and prevented the outflow from the opening after the puncture. The implanted pupae were put aside to check for possible haemolymph loss.

### 4.5. Statistical Analyses

Statistical analysis was performed with GraphPad Prism 6 statistical software (GraphPad Software, Inc., San Diego, CA, USA, PM license) using Mann-Whitney test. Significant changes were considered as those with a *p*-value of *p* ≤ 0.05 (*). A period of around 5 to 8 min in each 2-h period of each of the recording was assessed which correspond to adequate phase (anterograde and retrograde separately). The data are presented as average (± SD) values obtained from *n* replicates.

## Supplementary Materials

The following are available online at https://www.mdpi.com/2072-6651/11/5/287/s1, Table S1: Percentage changes in the heart beat frequency of *T. molitor* pupae during 24 h recordings including anterograde (A) and retrograde (R) phases. Red marked font presents the increase of heartbeats per minute, while blue marked font, the decrease. Presented data includes only statistically significant results (*p* < 0.05). Table S2: Percentage changes in the duration anterograde (A), retrograde (R) and diastasis (D) phases of *T. molitor* pupae heart beat during 24 h recordings. Blue colored font presents the phase reduction, while red font the phase elongation. Sign “-“ means lack of appearance of the phase. Presented data includes only statistically significant results (*p* < 0.05).
