# Supplementary material for: Differentiated Effects of Secondary Metabolites from Solanaceae and Brassicaceae Plant Families on the Heartbeat of Tenebrio molitor Pupae
Source: Toxins (Basel). 2019 May 22;11(5):287. doi: 10.3390/toxins11050287 (PMC6563514; doi:10.3390/toxins11050287)
Supplement: Supplementary file 1 [file toxins-11-00287-s001.pdf]

# Supplementary Materials: Differentiated Effects of Secondary Metabolites from Solanaceae and Brassicaceae Plant Families on the Heartbeat of *Tenebrio molitor* Pupae

**Paweł Marciniak, Angelika Kolińska, Marta Spochacz, Szymon Chowański, Zbigniew Adamski, Laura Scrano, Patrizia Falabella, Sabino A. Bufo and Grzegorz Rosiński**

**Table S1.** Percentage changes in the heart beat frequency of *T. molitor* pupae during 24 h recordings including anterograde (A) and retrograde (R) phases. Red marked font presents the increase of the heartbeats per minute, while blue marked font the decrease statistically significant ($p < 0.05$). Black color presents changes not statistically significant.

| Tested substances | Change of the heartbeat (%) | | | | | | | | | | | | | | | | | | | | | | | |
|---|---|---|---|---|---|---|---|---|---|---|---|---|---|---|---|---|---|---|---|---|---|---|---|---|
| | A | R | A | R | A | R | A | R | A | R | A | R | A | R | A | R | A | R | A | R | A | R | A | R |
| *S. tuberosum* extract | ↑ 5 | ↑ 1 | ↑ 14 | ↑ 14 | 0 | ↑ 17 | ↑ 38 | ↑ 29 | ↑ 40 | ↑ 17 | ↑ 29 | ↑ 67 | ↑ 20 | ↑ 35 | ↓ 12 | ↓ 13 | ↓ 14 | ↓ 1 | ↓ 14 | 0 | ↓ 17 | ↑ 3 | ↓ 19 | ↓ 2 |
| α-solanine | ↓ 54 | ↓ 3 | ↓ 6 | ↑ 7 | ↓ 19 | ↑ 38 | ↓ 6 | 0 | ↑ 23 | ↑ 23 | ↓ 15 | ↑ 13 | 0 | ↑ 49 | ↓ 18 | ↑ 10 | ↓ 1 | ↑ 24 | ↑ 2 | ↓ 38 | ↑ 22 | ↓ 38 | ↑ 4 | ↓ 77 |
| α-chaconine | ↑ 6 | ↑ 10 | ↑ 2 | ↑ 32 | ↓ 5 | ↑ 28 | ↑ 64 | ↑ 168 | ↑ 58 | ↑ 215 | ↑ 70 | ↑ 271 | ↑ 57 | ↑ 227 | ↑ 86 | ↑ 238 | ↑ 81 | ↑ 271 | ↑ 62 | ↑ 277 | ↑ 63 | ↑ 268 | ↑ 58 | ↑ 313 |
| *S. nigrum* extract | ↓ 11 | ↑ 24 | ↓ 4 | ↑ 8 | ↓ 5 | ↑ 20 | ↓ 3 | ↑ 22 | ↓ 3 | ↑ 18 | 0 | ↑ 16 | ↓ 1 | ↑ 28 | ↑ 5 | ↑ 17 | ↑ 1 | ↑ 22 | ↑ 6 | ↑ 34 | ↑ 3 | ↑ 23 | ↑ 10 | ↑ 31 |
| Solasonine | ↑ 10 | ↑ 13 | ↓ 9 | ↑ 65 | ↓ 2 | ↑ 91 | ↓ 15 | ↑ 15 | ↓ 5 | ↑ 73 | ↑ 59 | ↑ 15 | ↑ 50 | ↓ 18 | ↑ 43 | ↓ 28 | ↑ 12 | ↑ 121 | ↓ 15 | ↑ 88 | ↓ 10 | ↑ 68 | ↓ 4 | ↑ 123 |
| Solamargine | ↓ 25 | ↑ 69 | ↓ 27 | ↑ 112 | ↓ 29 | ↑ 118 | ↓ 29 | ↑ 71 | ↑ 33 | ↑ 75 | ↑ 21 | ↑ 49 | ↑ 24 | ↑ 47 | ↑ 23 | ↑ 72 | ↑ 41 | ↑ 18 | ↑ 42 | ↑ 20 | ↑ 43 | ↑ 68 | ↑ 52 | ↑ 48 |
| *S. lycopersicon* extract | ↑ 28 | ↑ 15 | ↑ 19 | ↓ 2 | ↑ 13 | ↑ 3 | ↑ 17 | ↓ 9 | ↑ 32 | ↓ 2 | ↓ 3 | ↑ 9 | ↓ 3 | ↓ 9 | ↓ 15 | ↓ 8 | ↓ 21 | ↓ 6 | ↓ 18 | ↑ 2 | ↓ 20 | ↑ 17 | ↓ 25 | ↑ 20 |
| α-tomatine | ↓ 8 | ↑ 16 | ↓ 12 | ↓ 12 | ↓ 8 | ↓ 4 | 0 | ↓ 33 | ↑ 3 | ↓ 12 | ↓ 13 | ↓ 11 | ↓ 14 | ↓ 24 | ↓ 13 | ↓ 24 | ↓ 17 | ↓ 9 | ↓ 6 | ↓ 7 | ↓ 22 | ↓ 11 | ↓ 10 | ↑ 38 |
| *A. rusticana* extract | ↓ 16 | ↓ 19 | ↓ 1 | ↓ 6 | ↓ 3 | ↓ 2 | ↓ 3 | ↓ 14 | ↑ 1 | ↓ 15 | ↓ 4 | ↓ 18 | ↓ 2 | ↑ 6 | ↓ 7 | ↓ 24 | ↓ 10 | ↓ 15 | ↓ 6 | ↓ 21 | ↓ 6 | ↓ 6 | ↓ 9 | ↓ 5 |
| Time (hours) | 2 | | 4 | | 6 | | 8 | | 10 | | 12 | | 14 | | 16 | | 18 | | 20 | | 22 | | 24 | |

**Table S2.** Percentage changes in the duration anterograde (A), retrograde (R) and diastasis (D) phases of *T. molitor* pupae heart beat during 24 h recordings. Blue colored font presents the phase reduction, while red font the phase elongation statistically significant ($p < 0.05$). Sign "-" means lack of appearance of the phase. Black color presents changes not statistically significant.

| Tested substances | Change of the phase duration (%) | | | | | | | | | | | | | | | | | | | | | | | | | | | | | | | | | | | |
|---|---|---|---|---|---|---|---|---|---|---|---|---|---|---|---|---|---|---|---|---|---|---|---|---|---|---|---|---|---|---|---|---|---|---|---|---|
| | A | R | D | A | R | D | A | R | D | A | R | D | A | R | D | A | R | D | A | R | D | A | R | D | A | R | D | A | R | D | A | R | D | A | R | D |
| *S. tuberosum* extract | ↓ 11 | ↑ 2 | ↓ 3 | ↑ 8 | ↑ 6 | ↑ 10 | ↑ 32 | ↓ 8 | ↑ 1 | ↓ 19 | ↑ 20 | ↓ 18 | ↓ 38 | ↓ 20 | ↓ 20 | ↓ 26 | ↑ 21 | ↓ 13 | ↓ 41 | ↓ 38 | ↓ 37 | ↓ 27 | ↓ 9 | ↓ 26 | ↓ 28 | ↓ 42 | ↓ 46 | ↓ 13 | ↓ 35 | ↓ 36 | 0 | ↓ 10 | ↓ 22 | 0 | ↑ 7 | ↓ 30 |
| α-solanine | ↓ 40 | ↑ 17 | ↓ 44 | ↓ 31 | ↓ 30 | ↓ 64 | ↓ 46 | ↓ 16 | ↓ 61 | ↓ 57 | ↓ 13 | ↓ 78 | ↓ 58 | ↓ 52 | ↓ 59 | ↓ 21 | ↑ 7 | ↓ 88 | ↓ 49 | ↓ 34 | ↓ 84 | ↓ 47 | ↑ 43 | ↓ 89 | ↓ 33 | ↓ 23 | ↓ 79 | ↑ 49 | ↓ 28 | ↑ 84 | ↑ 14 | ↑ 28 | ↑ 48 | ↑ 9 | ↑ 31 | ↓ 33 |
| α-chaconine | ↑ 33 | ↑ 10 | ↑ 19 | 0 | ↓ 14 | ↓ 16 | ↓ 49 | ↓ 13 | ↑ 20 | ↓ 60 | ↓ 69 | ↑ 328 | ↓ 54 | ↓ 66 | ↑ 295 | ↓ 28 | ↓ 11 | ↑ 139 | ↓ 42 | ↓ 42 | ↑ 116 | ↓ 32 | ↓ 43 | ↑ 118 | ↑ 30 | ↑ 14 | ↓ 14 | ↑ 43 | ↓ 12 | ↑ 28 | ↓ 1 | ↓ 5 | ↓ 10 | ↑ 25 | ↑ 7 | ↑ 13 |
| *S. nigrum* extract | ↑ 33 | ↑ 48 | ↓ 8 | ↑ 28 | ↓ 8 | ↑ 18 | ↓ 24 | ↓ 41 | ↑ 19 | ↓ 18 | ↑ 9 | ↓ 12 | ↓ 7 | ↓ 6 | ↓ 21 | ↓ 3 | ↑ 45 | ↓ 61 | ↑ 4 | ↑ 14 | ↓ 51 | ↑ 9 | ↑ 23 | ↓ 60 | ↑ 19 | ↑ 31 | ↓ 58 | ↑ 20 | ↑ 41 | ↓ 52 | ↑ 7 | ↑ 16 | ↓ 59 | ↑ 14 | ↑ 22 | ↓ 43 |
| Solasonine | ↓ 32 | ↑ 81 | ↓ 53 | ↓ 38 | ↑ 16 | ↓ 44 | ↓ 44 | ↓ 26 | ↓ 40 | ↓ 54 | ↑ 42 | ↓ 42 | ↓ 54 | ↓ 12 | ↓ 42 | ↓ 16 | ↑ 69 | ↓ 61 | ↓ 17 | ↑ 14 | ↓ 74 | ↓ 14 | ↑ 27 | ↓ 69 | ↓ 29 | ↑ 21 | ↓ 77 | ↓ 9 | ↑ 39 | ↓ 77 | ↓ 41 | ↑ 21 | ↓ 73 | ↑ 14 | ↑ 65 | ↓ 63 |
| Solamargine | ↓ 45 | ↑ 40 | ↑ 1 | ↓ 41 | ↑ 13 | ↓ 21 | ↓ 5 | ↓ 2 | ↓ 1 | ↓ 4 | ↓ 4 | ↑ 6 | 0 | 0 | 0 | 0 | 0 | ↓ 47 | 0 | 0 | ↓ 42 | 0 | 0 | ↓ 41 | ↑ 18 | ↓ 28 | ↓ 61 | ↑ 34 | ↑ 2 | ↓ 68 | ↓ 27 | ↓ 43 | ↓ 39 | ↓ 16 | ↓ 16 | ↓ 43 |
| *S. lycopersicon* extract | ↓ 13 | ↑ 5 | - | ↑ 56 | ↑ 18 | - | ↑ 29 | ↑ 23 | - | ↑ 28 | ↑ 41 | - | ↑ 12 | ↑ 14 | - | ↑ 2 | ↑ 17 | ↓ 14 | ↑ 9 | ↓ 11 | ↓ 1 | ↑ 1 | ↓ 2 | ↑ 57 | ↓ 4 | ↓ 10 | ↑ 26 | ↓ 1 | ↓ 9 | ↑ 39 | ↓ 14 | ↓ 10 | ↑ 26 | ↑ 17 | ↓ 7 | ↑ 53 |
| α-tomatine | ↑ 17 | ↓ 5 | ↓ 4 | ↑ 31 | ↓ 10 | ↑ 37 | ↓ 6 | ↓ 11 | ↑ 40 | ↑ 13 | ↓ 12 | ↑ 15 | ↓ 9 | ↓ 7 | ↑ 17 | ↑ 23 | ↑ 54 | ↓ 18 | ↑ 35 | ↑ 14 | ↓ 44 | ↑ 4 | ↑ 50 | ↓ 23 | ↑ 54 | ↑ 23 | ↓ 27 | ↑ 65 | 0 | ↓ 41 | ↑ 35 | ↑ 9 | ↓ 34 | ↑ 52 | ↓ 4 | ↓ 40 |
| *A. rusticana* extract | ↑ 46 | ↑ 12 | 0 | ↑ 45 | ↑ 1 | ↑ 30 | ↑ 12 | ↑ 8 | ↓ 3 | ↑ 13 | ↑ 10 | ↓ 10 | 0 | ↑ 7 | 0 | 0 | ↑ 22 | ↓ 30 | 0 | ↑ 13 | ↓ 28 | 0 | ↑ 29 | ↓ 10 | 0 | ↑ 8 | ↓ 23 | 0 | ↑ 20 | ↓ 5 | 0 | ↑ 13 | ↓ 9 | 0 | ↑ 18 | ↑ 7 |
| Time (hours) | | 2 | | | 4 | | | 6 | | | 8 | | | 10 | | | 12 | | | 14 | | | 16 | | | 18 | | | 20 | | | 22 | | | 24 | |
